# Obesity in Brazil: 214 million reasons to act

**DOI:** 10.20945/2359-4292-2026-0009

**Published:** 2026-02-16

**Authors:** Bruno Halpern, Maria Edna de Melo

**Affiliations:** 1 World Obesity Federation (WOF), London, UK; 2 Grupo de Obesidade, Departamento de Endocrinologia, Universidade de São Paulo, São Paulo, SP, Brasil; 3 Departamento de Advocacy, Associação Brasileira para o Estudo da Obesidade e Síndrome Metabólica (ABESO), São Paulo, SP, Brasil

World Obesity Day was established by the World Obesity Federation in 2015 and, since 2020, has been celebrated on March 4th as a strategy to raise awareness of the importance of unified actions to tackle the obesity epidemic, which is continuously growing worldwide, with no signs of reduction ^(1,2)^. This increase has been more rapid in low- and middle-income countries and, within countries, has been disproportionately higher among vulnerable populations and younger individuals ^(1,2)^.

In 2026, the campaign motto is “8 billion reasons to act”, referring to the current world population. Why is that? The World Obesity Federation (WOF) Atlas shows that, at this very moment, half of humanity lives with overweight or obesity, including more than one billion people with obesity itself ^(3)^. However, discussing and acting on obesity is a matter that concerns everyone. Obesity is associated with more than 200 diseases, increased disability and mortality, and major impairment in quality of life ^(2,4)^. Its impact extends beyond those who live with the disease, affecting society as a whole. As an example, the obesity epidemic imposes a substantial economic burden through direct healthcare costs and indirect costs such as work absenteeism ^(5)^, and it also impacts climate change and sustainability ^(6)^. Furthermore, providing healthy environments for people living with chronic diseases — especially children — is a human right that should be defended collectively.

In Brazil, as expected, the scenario is extremely concerning. The 2025 World Obesity Atlas showed that, for the first time, more than 30% of the population has obesity and 68% has at least overweight ^(3)^. According to WOF data from 2024, the projected annual growth rate in prevalence is 1.8% ^(7)^. Among children and adolescents, it is estimated that, in the absence of effective interventions, half will have excess weight by 2050 ^(7)^. Even under highly optimistic prevention scenarios, prevalence is expected to continue rising in the coming years and decades ^(8)^.

What can be done? First and foremost, it is essential to recognize that the obesity crisis requires actions in both prevention and treatment, not one instead of the other ^(2)^.

Brazil has one of the most internationally recognized and awarded Food-Based Dietary Guidelines, with a strong recommendation for “eating real food” and reducing the consumption of ultra-processed foods (UPFs) ^(9)^. For context, the concept of UPFs derives from the NOVA classification, developed in Brazil and now widely adopted internationally ^(10)^. High UPFs consumption has been repeatedly associated with at least 32 adverse health outcomes, far beyond obesity alone ^(10,11)^. In this issue of *Archives of Endocrinology and Metabolism*, Favaron and cols. provide cross-sectional evidence that adds to this body of literature ^(12)^. The authors show that a higher proportion of calories from UPFs is associated with poorer diet quality and more disordered eating patterns, including bulimia. Individuals in the highest tertile of UPFs intake presented higher scores for binge eating and bulimia symptoms, as well as greater emotional, external, and uncontrolled eating, and lower intake of minimally processed foods and protein. These findings suggest that UPFs may not only worsen nutritional quality but also be linked to neurobehavioral mechanisms that favor loss of control over eating, reinforcing maladaptive eating behaviors in obesity. Although similar associations have been described previously ^(13)^, a more comprehensive understanding of the relationship between UPFs, mental health, and disordered eating is still lacking. Nevertheless, the available data support the need for more active public actions to reduce UPFs consumption in Brazil.

However, implementing dietary guidelines and reducing UPFs intake is particularly challenging in vulnerable populations, in whom social and economic determinants strongly influence food choices ^(2,14)^. Unprocessed foods are often more expensive, whereas UPFs are cheap, ubiquitous, heavily marketed in traditional and social media, and easy to consume, requiring little or no preparation ^(10,14)^. As has already occurred in the United States and the United Kingdom, where UPFs account for approximately 60% of total dietary intake, the traditional Brazilian diet — based on home cooking and time for food preparation — is gradually being replaced by ultra-processed products ^(14)^. In Brazil, UPFs already contribute more than 20% of total energy intake, and this proportion is increasing every year ^(15)^.

Therefore, policies for obesity and other non-communicable diseases should prioritize structural public actions that increase the likelihood of healthy eating, rather than relying solely on generic recommendations for “better choices” without providing realistic conditions for change ^(2)^.

In 2022, the World Health Organization (WHO) launched the Acceleration Plan to Stop Obesity, and Brazil was identified as one of the front-runner countries ^(16)^. As with all health interventions, obesity prevention strategies should be guided by scientific evidence, not by opinion. The WHO highlights several policies that should be implemented, including taxation of sugar-sweetened beverages (SSBs) ^(16)^. During the Brazilian tax reform, taxation of UPFs was proposed; however, in 2024, an excise tax was approved only for soft drinks, after intense political debate and strong industry lobbying, not without many turnarounds. Considering the broad health impact of UPFs and the physiology of human eating behavior, which predisposes individuals to overconsume these products, fiscal policies should target far more than SSBs alone. Importantly, such strategies should not only increase taxes on unhealthy products — which could disproportionately affect vulnerable groups — but also reduce the cost of healthy foods, particularly fruits, vegetables, whole grains, and high-quality protein sources ^(16)^. The recent revision of the “*Cesta Básica*” (basic food basket — a list of essential foods for a minimum diet) introduced tax exemptions or reductions for some healthier foods; however, not all included items can be considered nutritionally adequate. Thus, although the tax reform represents a step forward, it remains modest and insufficient to produce a substantial population-level health impact ^(8)^.

The WHO also recommends additional measures, such as restricting the marketing of unhealthy foods, especially to children and adolescents; improving front-of-package labeling; strengthening school nutrition policies; and modifying the built environment to reduce physical inactivity ^(2,16)^. Several of these strategies have been partially implemented in Brazil, but their effectiveness has been limited. For example, the rapid growth of social media use among younger populations has not been accompanied by adequate regulation of food marketing on digital platforms, a challenge that extends beyond Brazil ^(17)^.

An integrated and unified strategy addressing all these fronts simultaneously is essential, as isolated measures are unlikely to be effective, particularly given the food industry’s capacity to rapidly adapt and circumvent regulations ^(14)^.

The WOF is currently conducting the MAPPS II programme, which explores the perceptions and experiences of healthcare professionals, policymakers, and people living with obesity regarding existing policies, gaps, and priorities. This initiative aims to strengthen obesity prevention and care through improved evidence generation, coalition building, and policy engagement.

Nevertheless, even the most effective prevention strategies will not solve the problem for those who already live with obesity, just as condom distribution alone does not address the needs of people already living with HIV ^(2)^. Therefore, advocacy for people living with obesity (PlwO) is crucial, despite the barriers imposed by structural stigma. In Brazil, ABESO has advanced this agenda by creating a “Lived Experience” Department and promoting the inclusion of PlwO in public health discussions and decision-making spaces.

Within the Brazilian Unified Health System (SUS), the only available treatment for obesity is bariatric surgery. Although highly effective and associated with substantial improvements in health and quality of life, it is restricted to individuals with severe obesity (class III or class II with comorbidities), is not scalable, and is offered to fewer than 10,000 patients per year, resulting in extremely long waiting lists. Moreover, surgery is indicated only after failure of clinical treatment, despite the fact that structured clinical care is largely unavailable. Postoperative nutritional supplementation is also not routinely provided, despite the known risk of micronutrient deficiencies ^(18)^.

Over the years, several proposals to incorporate obesity medications into the public system have been submitted, many led by ABESO, but all were rejected by the National Committee for Health Technology Incorporation (CONITEC), even in the presence of robust cost-effectiveness data. Non-pharmacological standards of care are also scarce, and the situation is aggravated by the lack of formal education in obesity within health professional training programs. As a result, most healthcare professionals, including physicians, graduate without adequate training in obesity, limiting their ability to provide effective and non-stigmatizing care.

With the growing evidence of the broad health benefits of modern obesity treatments — well beyond weight loss — and the publication of the WHO Guidelines on glucagon-like peptide-1 (GLP-1) receptor agonists for obesity ^(19)^, it is inevitable that pharmacological therapy will eventually be incorporated into SUS, at least for selected populations. This should occur sooner rather than later. The obesity crisis is not a problem of the future ^(2,3)^; it has been a reality for decades, and in Brazil alone there are more than 214 million reasons to act now.

## Data Availability

datasets related to this article will be available upon request to the corresponding author.
